# Some Later Herbalists

**Published:** 1894-10-20

**Authors:** 


### The First Century of English Medical Literature.

### IX.—SOME LATER HERBALISTS.

Among the earliest Englishmen who spent their energies n the endeavour to unveil the secrets of nature, William Turner's name is conspicuous. Educated at Pembroke Hall, Cambridge, of which College he was a Fellow, he early devoted himself to the study of plants, especially from a medical point of view, and in 1535, while still in residence at Cambridge, he compiled his first contribution to botanical knowledge, a small Latin herbal, of which no copy seems to have survived. He was even at this time, and always after, an ardent member of the reformed faith, and complains bitterly of the "crafty, covetous, and Popish printer," who in giving his book to the public, suppressed both the name and preface of the author. At the time when this early production was issued, Turner informs us that there was neither Greek, Latin, nor English name of tree or herb to be learned even of physicians; that only one English herbal existed (the Great Herbal), and this the young student had already perceived to be full of mistakes. The great Continental botanists, Fuchsius and Matthiolus, had not yet produced their learned works, when the young Turner, moved by a like spirit of patient investigation, set himself to enlarge and verify his knowledge. His next step was the publication, in 1548, of a useful compendium, probably put together originally for his own convenience, of the names of all known herbs, in Greek, Latin, English, Dutch, and French, with the additions of the familiar names ascribed to them in herbaries and apothecaries' use. This work is dedicated to Edward, Duke of Somerset, Protector of the Realm, to whom Turner had now become attached in the quality of private physician. In Somerset's house at Spon Syon Turner remained some considerable time. Here he planned the scheme of his great later work, delaying its commencement, however, till he could visit the West of England and make personal observations on the herbs to be found there. During this time of Somerset's protection and favour Turner enjoyed many opportunities of seeing and conversing with the Princess Elizabeth, the conversations between the young Princess and the physician being carried on with a quaint pedantry in Latin. Nearly twenty years after in dedicating his Herbal to the Queen, Turner recalls to her memory this season of classic intimacy, assuring her "that as for your knowledge in the Latin tongue, I had in the Duke of Somerset's house a good trial thereof, for I have both in England, low and high Germany, and other places of my long travel and pilgrimage never spake with any noble or gentlewoman that spake so well, and so much congrue fine and pure Latin as your Grace did unto me so long ago." The long travel and pilgrimage doubtless began on the death of Edward VI. Previous to that event, which dashed the hopes of all in favour at the Protestant Court, Turner seems to have been induced to take holy orders, and was installed as Dean of Wells. He held his Deanery under no very secure tenure, since he renders thanks to Elizabeth that four times with letters patent she had aided in restoring him to his cathedral. During the time of his exile he travelled in Italy and Germany, botanising everywhere, and corresponded with Fuchsius, and other learned foreigners. He acknowledges his great debt in particular to Italian writers on botany. Finally from his house in the Crossed Friars, 1568, he issued the Herbal which he had meditated from his youth, and which is well worthy to stand as his life work. Beautifully and delicately illustrated, the flowers and herbs are described with a wealth of language and epithet, which everywhere testifies to his love of nature. The medical part is evidently of a matter of minor importance to him. For the "vertues" of the herbs, he goes to Dioscorides, Mesue, Galen, Pliny, &c. For the descriptions no eye has been trusted but his own, and every petal and leaf is figured with an accuracy of detail which testifies to the loving care of the observer. The third part of Turner's Herbal was issued from the Deanery of Wells. In a letter which he prefixes to the Worshipful Company and Fellowship of Surgeons, he speaks of himself as vexed with sickness and the cares of his Deanery. Yet he is struggling to make the great work as perfect as possible, and has chiefly still at heart the advancement of physic and surgery by rendering available a right knowledge of the simples, which it is clear he loved quite as much for their own sakes as for their uses.

The most formidable rival to Turner's Herbal in this century was issued in 1578, and was a translation of the new Herbal, or "History of Plants," by Rembart Dodeon. The principal excellence of the work lies in its extraordinary thoroughness and accuracy of method. It has none of the poetry of Turner's writing, and it lacks all the little touches of local observation which raise the Dean's descriptions to so high a pitch of excellence. As a book of reference Dodeon might conceivably prove the more useful. His list of "vertues" is derived from much the same authorities and presents no substantial variations.

From this learned work was compiled, in 1606, a small popular treatise on which the odd title of "Ram's Little Dodeon" was bestowed. It was intended to bring Dr. Dodeon down to the people, and professes to give his practice on one side of the page and on the other those of various practitioners, probably of Ram himself. There is a great deal more of Ram than of Dodeon in the curious jumble of herbs which are prescribed for every ill. The following prescription will serve as a good specimen of the knowledge of simples displayed in it. It is for sore eyes—"if a man had lost his sight ten years, it shall heal in ten days, *if possible*." "Smallache, rue, fennell, varnen, egrimony, belony, avens, scabious, handstrong, enfrase, pimpernel, and sage, a like much; distil them with a little urine of a man child with five grains of frankincense—a drop every night in a sore eye."

A medical work relying more exclusively on herb remedies, written by William Langham, practitioner in physic, under the title of "The Garden of Health," 1578, must not be omitted. The terrible extent to which hydrophobia was prevalent in those days may be gathered from the fact that no fewer than thirty-three remedies are proposed for it, including such easy antidotes as bread, thistles, walnuts, wheat, figs, and maidenhair fern. But William Langham is worth remembering for his cure for melancholy; the prescription is no less than to rub the body all over with nettles, while those oppressed with "fear and fancy" are to carry nettles in their hand—a bracing procedure, no doubt, and one in which may perhaps be traced the first dawnings of homœopathic theory.

